# Perceived Stress Among Different Occupational Groups and the Interaction with Sedentary Behaviour

**DOI:** 10.3390/ijerph16234595

**Published:** 2019-11-20

**Authors:** Audrius Dėdelė, Auksė Miškinytė, Sandra Andrušaitytė, Žydrūnė Bartkutė

**Affiliations:** Department of Environmental Sciences, Faculty of Natural Sciences, Vytautas Magnus University, Vileikos Street 8, 44404 Kaunas, Lithuania; aukse.miskinyte@vdu.lt (A.M.); sandra.andrusaityte@vdu.lt (S.A.); zydrune.bartkute@vdu.lt (Ž.B.)

**Keywords:** physical activity, sedentary behaviour, perceived stress, occupation, white-collar, blue-collar

## Abstract

Sedentary lifestyle and low physical activity are associated with health issues, including both physical and mental health, non-communicable diseases, overweight, obesity and reduced quality of life. This study investigated differences in physical activity and other individual factors among different occupational groups, highlighting the impact of sedentary behaviour on perceived stress by occupation. Cross-sectional study included 571 full-time workers of Kaunas city, Lithuania. The outcome of this study was assessment of perceived stress. Time spent sedentary per day, occupation and other individual characteristics were self-reported using questionnaires. Two main occupational groups were analysed: white-collar and blue-collar workers. Multivariate logistic regression was used to assess the impact of sedentary behaviour on perceived stress among different occupational groups. The prevalence of high sedentary behaviour was 21.7 and 16.8 % among white-collar and blue-collar workers, respectively. Blue-collar workers had a higher risk of high perceived stress (OR 1.55, 95% CI 1.05–2.29) compared to white-collar workers; however, sedentary time did not have any impact on high perceived stress level. Meanwhile, white-collar male (OR 4.34, 95% CI 1.46–12.95) and white-collar female (OR 3.26, 95% CI 1.23–8.65) workers who spend more than three hours per day sedentary had a greater risk of high levels of perceived stress. These findings indicate sedentary behaviour effect on perceived stress among two occupational groups—white-collar and blue-collar workers—and other important factors associated with perceived stress.

## 1. Introduction

### 1.1. Work Stress and Health

Stress is an inevitable part of today’s fast-paced life and the way stressors are being managed can have a great impact on mental and physical health and overall life quality. Due to increasing working hours and the demands and pressure to succeed [1], work can become one of the most challenging environments for people to handle. There are many different stress sources at the workplace: those intrinsic to the job; those related to the role in the organization, career development, relationships at work, organizational structure and climate [2].

Some professions are believed to be more emotionally demanding and stressful than others. Ambulance workers, teachers, social service workers, customer service and call centre workers, prison and police officers are found to be most physically and psychologically demanding, occupations, while the people in these professions are also least satisfied with their jobs. The least stressed and most satisfied are those working as analysts, school lunchtime supervisors and directors in the private sector [3]. 

Professions where communicating and helping others are the key components of work are thought to be the most emotionally demanding as a result of the psychological strain that carries onto non-working time, affects sleep and reduces recovery from work stress and/or fatigue [4]. Healthcare workers are two times more likely to get depression compared to engineering, architecture and surveying occupations [5]. 

The same stressor can have a completely different effect on different individuals and cause diverse health risks [6,7]. Both acute and chronic stress can have long-term consequences on cardiovascular, metabolic, immune systems and the brain (memory problems, aging and cognitive impairment) [6]. Stress-related work is associated with elevated blood pressure (BP), digestive system disorders, depression, anxiety, use of alcohol and drugs, back pain, headache, eye strain, sleep disturbance, dizziness and fatigue [8]. Workers who suffer from depression and anxiety are less likely to engage in sports and overall physical activity [9] and spend less time in total performing moderate to vigorous physical activities [10]. Employees with depression and experiencing high levels of stress were estimated to be 48% and 9% more expensive (or by $2,000 and $413 higher costs) respectively, on average per person annually [11].

### 1.2. Occupational PhysicalActivity and Sedentary Behaviour

Although physical activity is one of the basic human functions, contrary to this, sedentary behaviour is increasing worldwide [12,13,14]. Physical activity is considered as one of the most important factors positively influencing both physical and mental health, preventing cardiovascular diseases through strengthening haemodynamic, neuroendocrine, inflammatory and haemostatic responses to mental stressors [15]. Physical activity lowers the risk of diabetes, breast and colon cancer, depression [12], eliminating between 6 % and 10 % of the major non-communicable diseases (NCDs) [16]. Lack of physical activity has a major health impact and leads to falls, overweight, obesity and has negative effect on the quality of life. A global study carried out from 2001 to 2016 representing 96 % of the global population showed that more than a quarter of all adults were not getting enough physical activity [12]. A survey carried out in the 28 European Union Member States in 2017 showed that nearly half (46%) of Europeans never exercise or play sports and this proportion has been increasing gradually in recent years [17].

The World Health Organization’s [18] recommendations on physical activity for adults (≥18 years) is 150 min of moderate-intense activity (or equivalent) per week, performed in multiple domains: work, travel (walking and cycling) and recreation (including sports). WHO has set a goal to reduce physical inactivity 10% by 2025 and 15% by 2030.

The type of work environment and methods, profession and its main functions play an important role in everyday life. Physically active jobs are significantly different from sedentary types of jobs, especially as far as hourly steps and the heart rate are concerned [19]. Men with high occupational physical activity and low leisure time physical activity have an increased risk of cardiovascular disease and all-cause mortality [20]. Those employees who often lift, carry heavy loads and work with hands above the shoulder are observed to have increased systolic BP at work, at home and during sleep [21]. High level of moderate to vigorous leisure time physical activity significantly lowers BP during daytime at work and at home [21].

Regarding gender, men working full-time and even sedentary jobs are more active than healthy non-workers. On the contrary, women with full-time sedentary jobs spend more time sedentary and have a less intensive activity than healthy non-workers [22].

Sedentary behaviour is described as energy expenditure at 1.0–1.5 metabolic equivalent units (METs) [23,24]. The common examples of sedentary behaviour are TV watching, computer or other screen-based use and/or viewing and desk-based work. Sitting dominates in all of these activities, so it can be referred to as ‘sitting time’ [23].

Due to the growing use of technologies, sedentary lifestyle is taking one of the biggest parts of workers’ day. It was estimated that young adults could spend up to 9.2 h/day sedentary [14,25]. 

Daily occupation-related energy expenditure has decreased by more than 100 calories over the last 50 years and this accounts for a significant increase in average body weight for both women and men. Five decades ago, almost half of the jobs required at least moderate intensity physical activity; nowadays it is less than 20% [13].

Physical inactivity is one of the obstacles that workers have to face when trying to adopt healthy lifestyles in their work areas. It is considered as important as poor diet, smoking, illnesses and lack of knowledge [26] because physically inactive employees are 15% more expensive in comparison with physically active employees as far as increased medical costs are concerned [11].

Managers, other white-collar workers and students are more likely to spend more than 8 h per day sitting down, but these occupational groups usually perform more light activities in comparison with blue-collar workers, who participate more in moderate and high intensity activities and also accumulate lower levels of occupational sedentary time [27,28]. Workers with desk jobs (professionals, managers and administrative workers) that have the greatest number of hours spent sitting at work, are found to have the lowest number of steps on weekdays. Workers who spend the lowest number of hours sitting at work (technicians and blue-collar workers), record the highest number of weekday steps. A review study by Smith et al. [28] identified that being in full-time employment and being an older age were positively associated with occupational sedentary behaviour, meanwhile having a blue-collar occupation and smoking were negatively associated with sedentary behaviour. There is also evidence to suggest that non-occupational sitting time and screen time (TV viewing, computer use) may be adversely associated with mental well-being in employed adults [29]. Findings from this study indicated potential moderating effect of gender on associations between sedentary behaviour and mental well-being.

This study aimed to examine the relationships of sedentary behaviour outside work, demographic, socioeconomic, behavioural and health factors among white-collar and blue-collar workers as well as to assess perceived stress levels among the aforementioned groups of workers. 

The present study is different from other similar ones due to its focus on two occupational groups—blue- and white-collar workers. These are the two most common occupational groups in the working society, so the results do not only describe the current situation and existing relations, but also provide insights into how much the type of work influences our lifestyle choices and forms our habits. Most of the previous studies analysed and compared specific occupational groups which in most cases were monitored/classified only by physically active/sedentary type of jobs (e.g., brewery workers versus call centre/office workers) [19], or analysed the association of occupational and leisure time physical activity with the risk of all-cause mortality [20]. Unlike other studies in the relevant literature, this study not only investigates physical activity and/or sedentary behaviour [27] and stress/mental health of certain occupational groups separately [3,6], but also combines these two aspects—sedentary behaviour and stress—and investigates their currently existing relations. The purpose of this study is to contribute to decreasing the levels of sedentary behaviour and stress and their negative impact on health. Therefore, this study aims to answer how blue- and white-collar workers perceive stress and how this relates to sedentary behaviour. Since sedentary or physically active jobs cannot be changed very easily or switched from one to the other because it is part of the specific work requirements and routine, changing other lifestyle habits and adjusting health choices can lead to better stress management and better quality of life.

In addition, we examined different characteristics (demographic and socioeconomic factors, the use of travel modes, smoking, body mass index (BMI), chronic disease and hypertension (HP)) among blue- and white-collar workers, with sedentary behaviour and physical activity being the most important ones and investigated potential risk factors for stress. The findings of the present study could be applied to other European countries, particularly the population of the Baltic region, especially for further investigation with an objective outcome measurement in the future.

## 2. Materials and Methods 

### 2.1. Study Design and Participants

The study was a cross-sectional survey which was conducted in 2017 by a research market agency and designed based on telephone interviews. Participants for the study were selected through random sampling to represent the entire population of Kaunas city, Lithuania. Among the participants, 571 full-time workers were included in the study to investigate the relationships between sedentary behaviour, perceived stress and occupational groups. The study received ethical approval from the Kaunas Regional Biomedical Research Ethics Committee (Approval No. BE-2-16).

### 2.2. Assessment of Perceived Stress and Sedentary Behaviour

Perceived stress was investigated by a questionnaire based on the Reeder stress scale [30] which is commonly used to measure stress and has been validated in a cross-sectional study in the United Kingdom [31]. Seven statements experienced in everyday stressful situations were used to evaluate perceived stress levels of the study participants as “usually tense or nervous”, “work-related concerns”, “daily activities are extremely trying and stressful”, “feeling nervous”, “nervous strain related to my daily activities”, “tense when communicating with other people”, “physically and mentally exhausted by the end of the day”. Individuals had to indicate the extent to which each statement applied to them: exactly, to some extent, not very accurately, or not at all. Each of the seven item responses was scored from 1 (strongly agree) to 4 (strongly disagree) and an overall score was calculated by summing up the seven individual item scores. A scoring system was used to derive a summary score ranging from 7 (high perceived stress) to 28 (low perceived stress). Cronbach’s alpha coefficient for the stress scale was 0.78 (good).

In the study, the participants were asked “How many hours per day on average did you spend sitting outside of work?“. They were asked to estimate in total the number of hours per day they spent sitting on a weekday and a weekend day, not including the time spent sitting at work. The study participants were then divided into two groups according to the median value: 1) less than three hours of sedentary behaviour per day and 2) three hours or more of sedentary behaviour per day [32]. Sedentary time was dichotomised based on the median value of our study sample; however, the analysis of other studies showed that non-work-related sitting time in front of the TV or computer (screen time) of our study sample was very similar to that of other studies. According to the statistical office of the European Union (Eurostat), the results from a survey carried out in 15 EU countries among adults (the age group 20 to 74) between 2008 and 2015 showed that the time outside work that adults spend on screen-related activities ranged from 3 h 17 min in Belgium and 3 h 14 min in Greece to 2 h 18 min in Italy.

### 2.3. Covariates

Demographic and socioeconomic factors, information about occupational groups, occupational and leisure-time physical activity behaviour, sedentary behaviour, the use of travel modes, perceived stress, smoking, body mass index (BMI) and health factors, such as chronic diseases and hypertension (HP), were obtained using questionnaires.

Educational level was divided into three groups: low, medium and high. Two income groups were distinguished: (1) ≤ €1000 and (2) > €1000.

Occupational and leisure-time physical activities were classified into three groups: low, moderate and high. Levels of physical activity (min/week) were divided into two groups according to the WHO physical activity recommendations: (1) less than or equal to 150 min/week and (2) more than 150 min/week. Participants were divided into median according to sedentary behaviour: (1) less than 3 h/day (< median) and (2) more than or equal to 3 h/day (≥ median).

Participants were classified into two groups according to their smoking status as follows: (1) non-smokers and (2) smokers. Participants’ height and weight measures were used to calculate BMI (kg/m^2^). According to the BMI categories, those with a BMI less than 25 were classified as normal weight, those with a BMI between 25 and 29.9 were classified as overweight, and those with a BMI over 29.9 were classified as obese. Two BMI groups were distinguished: (1) normal weight and (2) overweight/obesity.

The use of travel mode for the daily commute was classified into four groups: (1) those who walk, (2) public transport users, (3) those who ride a bike and (4) car users.

Occupations were classified according to the International Standard Classification of Occupations (ISCO) and grouped into white-collar (ISCO-88 major occupational groups 1–5) and blue-collar (ISCO-88 major occupational groups 6–9) workers [33]. According to the collar type of the occupation, white-collar workers are those who perform professional, managerial or administrative jobs, typically in an office or other administrative setting. Blue-collar workers include those who work in hard manual labour and in many other types of physical work.

### 2.4. Statistical Analysis

Descriptive statistics were used to identify frequencies and percentages of sample characteristics. The chi-square test and its *p*-value were calculated to determine the relationship between two categorical variables. Phi and Cramer’s V correlation coefficients were used to determine the strength of the association between categorical variables; the phi coefficient was used for two nominal variables, each of which had only two categories, and Cramer’s V was used for one nominal variable with either another nominal variable, or with an ordinal variable. The phi coefficient ranges from −1 to +1, with negative numbers representing negative relationships. Cramer’s V varies from 0 to 1. The closer the value is to 1, the stronger the linear relationship is between the two variables.

Binary logistic regression was applied to determine the association between occupational groups, sedentary behaviour and perceived stress by gender. In the model, the covariates included were gender, income, children (<18 years), BMI, work PA, marital status, smoking and chronic diseases. Covariates that were significantly associated with perceived stress (*p* < 0.05) or changed the adjusted odds ratios (aOR) by 10% or more were included in a multivariate logistic regression analysis.

Statistical analyses were performed using SPSS version 25.0 (released 2017. IBM SPSS Statistics for Windows, Version 25.0 IBM Corp.: Armonk, NY, USA).

## 3. Results

### 3.1. The Relationship Between Participants’ Characteristics and Occupational Group

The mean age of the study population was 42.3 (±11.0) years, 51.9% were female and 44.1% of the participants had high level of education. 

In Table 1 frequencies and percentages (%) of participants’ characteristics and p values of Χ^2^ tests and a correlation for categorical variables are presented. Participants were classified by occupational group (white-collar vs blue-collar). The results showed significant age differences between the different occupational groups (*p* = 0.002). The highest percentage of white-collar workers was determined in the youngest age groups (≤ 30 and 31–45 years). Meanwhile, the results were opposite with blue-collar workers, with the highest percentage in the oldest age groups (46–60 and ≥ 61 years). Gender difference between the two occupational groups was statistically significant (*p* < 0.001). The percentage of women (65.2%) was higher than that of men (34.8%) in the white-collar group, while among the blue-collar workers a higher percentage of men (88.1%) than that of women (11.9%) was determined. The differences emerged among occupational groups and educational level. More than half (54.2%) of the white-collar workers had the highest level of education. Meanwhile, 64.3% of the blue-collar workers had low levels of education. The levels of occupational and leisure-time physical activity were different between white-collar and blue-collar groups (*p* ≤ 0.001). The results showed that a higher percentage of both white-collar (56.2%) and blue-collar (70.5%) workers was detected in the higher income group. Statistically significant differences between white-collar and blue-collar groups were determined with regards to smoking and BMI. The prevalence of smoking (67.8%) and overweight/obesity (73.3%) was higher among the blue-collar workers compared to the white-collar workers (25.2 % and 51.0 %, respectively). On average, 70.1% of white-collar and 81.8% of blue-collar workers used a car for their daily commute. We found that among both white-collar (53.0%) and blue-collar (63.6%) workers there was a higher percentage of such individuals who reported high perceived stress.

### 3.2. The Relationship Between Participants’ Characteristics and Perceived Stress

Table 2 shows frequencies, percentages and odds ratios with 95% confidence intervals that indicate the association between individual characteristics and stress level. The results showed that the level of perceived stress increased with increasing age (Table 2). Moderate-intensity PA at work was associated with lower perceived stress level (OR 0.48; 95% CI 0.32–0.71). Meanwhile, a low level of physical activity per week (≤150) was significantly associated with a higher perceived stress level (OR 2.97; 95% CI 1.27–6.94). Workers with higher education had a tendency for lower levels of perceived stress. Our findings showed that overweight/obesity increased the risk of the higher level of perceived stress (OR 2.62; 95% CI 1.79–3.84). We found that higher perceived stress was associated with the increased risk of chronic diseases (OR 3.53; 95% 2.17–5.76) and HP (OR 7.35; 95% 3.28–16.45). Active mobility, such as cycling, has a positive effect on the perceived stress level. Bicycle users had 68% (OR 0.32; 95% 0.11–0.92) lower perceived stress risk compared to those individuals who reported not riding a bike for their daily commute. However, a very small number of participants were obtained in our study with higher levels (at least 150 min/week) of PA. Our findings revealed that the blue-collar workers perceived 55 % (OR 1.55; 95% 1.05–2.29) higher stress compared to the white-collar workers. We treated these variables as possible risk factors for perceived stress.

### 3.3. The Relationship Between Sedentary Behaviour and Perceived Stress Level by Occupational Groups

Table 3 shows the results of the multivariate logistic regression models analysing the relationship between perceived stress and sedentary behaviour among different occupational groups stratified by gender. Sedentary behaviour among the white-collar workers with reference to the group of sedentary behaviour less than 3 h per day was found to be a significant risk factor for perceived stress in males (aOR 4.34; 95% CI 1.46–12.95) and females (aOR 3.26; 95% CI 1.23–8.65), as shown by the multivariate model adjusted for incomes, children (<18 y), BMI and work PA. Our findings showed that sedentary behaviour among the blue-collar workers had no impact on high perceived stress in both women and men.

## 4. Discussion

This study examines the relationships between sedentary behaviour and perceived stress among two main occupational groups—white- and blue-collar workers. One of the biggest strengths of this study is that occupational groups are being compared by what impact different health risk factors and behaviour have on stress.

The white-collar male and female workers of our study had more than 4 and 3 times greater risk of high perceived stress, respectively, when spending more than 3 h per day sedentary (out of work) compared to the same occupational group that spent less than 3 h per day sedentary. Sedentary time of the blue-collar workers did not have any impact on the high perceived stress level. White-collar workers, compared to blue-collar workers, are more satisfied with their achieved career position, but it requires higher levels of decision latitude [34], so stressful and tense situations may be inevitable. We found that the white-collar workers were almost 2.5 times less actively participating in the high level of physical activity at the workplace than the blue-collar workers. The findings of this study suggest that when most of the workday for the white-collar workers contains sedentary passive behaviour, more than 3 additional hours spent in a sitting position per day can trigger a stress process. It was found that even the type of the workplace can greatly influence physical activity with workers in open bench seating being the most active, followed by workers in cubicles and private office workers being the least active [35]. There is evidence that more physically active workers had lower stress levels outside of work [35].

White-collar workers are less physically active and perform more light and low strain physical activities at the workplace in comparison with blue-collar workers, which was also acknowledged by other researchers [27,28]. This outcome was expected since 70% of blue-collar workers perceive their job as physically demanding [34]. It was suggested that team leaders and people in other high-decision latitude job positions and employees suffering from stress and tension could achieve better regeneration of physical and psychosocial resources by practicing physical fitness [18].

In our study, the blue-collar workers had 1.5 times higher risk of high level of perceived stress compared to the white-collar workers. This may be one of the reasons why the blue-collar workers had a higher tendency to smoke and had a higher BMI or were obese. Stressful living is closely associated with unhealthy lifestyle habits. Syamlal et al. [36] found that some occupations had the highest odds of current smokers. These included male labourers and females who worked in services occupations. These workers were found to have lower levels of education and income and were more vulnerable socially. Blue-collar workers compared to white-collar workers usually face bigger challenges because their job positions require continuous physical activity (construction, logging, fire-fighting), with an increasing risk of cardiovascular overload, fatigue and musculoskeletal disorders [19]. Work fatigue is strongly associated with work overload, lack of vacation or leisure time and frequent overtime hours, conflicts with the boss or colleagues [37]. Workers with high job strain and high effort-reward imbalance have a twofold higher risk of death from cardiovascular diseases [38] and type 2 diabetes [39]. Low job control is also mentioned as one of the most important factors of work stress [7]. Stressful work circumstances may eventually cause anxiety and depression. Melchior et al. [40] found that workers who are exposed to high psychological job demands have a two times higher risk of depression and anxiety than those with low demands. All these reasons make blue-collar workers more physically and physiologically vulnerable in comparison with white-collar workers.

This study showed that those participants who chose to cycle for the daily commute had 68% lower risk of high perceived stress, however the number of individuals who commuted to work by bike was small and this may have affected the strength of the association. This outcome is also supported by other researches: a study performed in the United Kingdom in 1991–2008 found that cycling and walking had a positive impact on the psychological wellbeing [41]. Hadgraft et al. [42] concluded that a higher level of leisure physical activity, cardiorespiratory fitness and body composition (lower BMI and body fat percentage) are associated with lower stress levels during work hours and throughout the day. Physical activity positively influences emotional well-being and, in this way, contributes to workers’ productivity [35]. Cycling to work was not popular among our study respondents (~3%), so there are many opportunities left to improve this behaviour. National habits, common traditions, developed infrastructure and climate conditions have a great impact on choosing different ways to reach the workplace. It was found that bike facilities, interesting things to look at, walkable access to transit stops, crime rate and overall walkability are the most important built environment features that significantly support workers’ physical activity in the workplace neighbourhood [43].

Regarding leisure PA, the white-collar workers of our study were more willing to participate in moderate intensity physical activity than the blue-collar workers (67.1 % versus 49.0%; *p* ≤ 0.001). This is a favourable tendency because desk workers should compensate sedentary behaviour by improving leisure time physical activity. Researchers have suggested that desk workers should accumulate additional 2000–3000 steps a day [25] or even more—60–75 min per day high level of moderate intensity physical activity to eliminate the increased risk of mortality associated with high sitting time (>8 h) [44].

Overall, we found that perceived stress was closely associated with health risk factors. The respondents of both occupational groups who were not physically active enough (PA min/week ≤150) had almost a 3 times bigger risk to experience the high level of perceived stress. However, there was a small number of participants who reported reaching higher levels (at least 150 min/week) of PA and this could reduce the statistical power and lead to overestimation or underestimation of true effect. 

The most significant odds ratio of all risk factors was found between HP and high perceived stress (OR 7.35; 95% 3.28–16.45), which shows that there is the association between cardiovascular health and everyday stress. This was also acknowledged by other authors [8]. Clays et al. [21] found that high level of moderate to vigorous leisure time physical activity lowers daytime BP and can help to prevent and control HP. Since respondents already had HP during our study, we suggest to take care of stress levels by trying to achieve WHO physical activity recommendations in order to manage this condition. Another statistically significant association was found between chronic diseases (OR 3.53; 95% 2.17–5.76), overweight/obesity (OR 2.62; 95% CI 1.79–3.84) and the higher perceived stress. These findings are consistent with previous studies that show that older age (which leads to an increasing risk of chronic diseases) and higher BMI were both associated with higher office stress levels [35]. Regular physical activity can help to control weight gain and obesity, which is a significant risk factor for high levels of stress.

Active lifestyle could help manage stress control and ease off its negative consequences to the overall health. Physical activity promotion, starting from active forms of commuting, could play a big part in workers’ everyday life activity. 

Simple interventions to increase activity at work—climbing the stairs, standing up more, walking during breaks, performing some stretching exercises or even introducing some special physical equipment, such as treadmills, exercise bikes, showers, other exercising facilities and adjusting the workplace accordingly,- have been shown to significantly impact both physical and work performance and increase daily activity caloric expenditure [45].

This study has some limitations that could be enhanced in future studies. The main limitation of this study is the lack of objective measures of sedentary behaviour outside of work and physical activity. Self-reported occupational and leisure-time physical activity as well as sedentary time could be subject to bias and may lead to underestimation of the strength of some associations between activity and risk factors [46]. There is evidence of lack of agreement between self-reported and objectively measured time spent sitting among workers [47,48,49], which could lead to underestimation of sedentary time. However, most of other studies investigated sedentary behaviour at work, whereas we studied sedentary behaviour outside work. The study conducted in the UK [47] showed that the type of assessment of self-reported sedentary behaviour influenced the measurement characteristics and that proxy measures, such as TV time used to assess the time spent sitting, showed the best precision compared to the objective measure. Similar results are found in studies examining differences between self-reported and objectively measured physical activity. Studies conducted among Canadian adults [50] and among the blue-collar workers in Denmark [51] showed a modest correlation between self-reported and accelerometer-measured physical activity, but associations with health markers existed between both physical activity variables [50]. However, most studies on physical activity have examined the general adult population and there are only a few studies on self-reported and measured physical activity among full-time workers which mainly focused on occupational physical activity. Another limitation of this study is a small number of groups for some independent variables such as the higher level of PA, bicycle commuters and walkers. Therefore, further studies with larger sample size are needed to confirm these results. 

It should also be noted that questionnaires are the most accessible and commonly used method for studies of a large population because it would be too complex and time-consuming to objectively measure physical activity with wearable devices in a large population [52,53].

The data on perceived stress were obtained from a questionnaire, which could result in the misclassification of the outcome and could attenuate the strengths of the observed associations. The study on the effects of the physical work environment on work-related stress in the U.S. suggests that work stress may affect some of the physiological responses (such as heart rate variability, salivary cortisol) associated with the negative health effects without the individuals being consciously aware of a stressful experience [54]. In that case, the difference between the physiological stress response and perceived stress is determined. However, the findings of studies examining the differences between physiological and self-reported stress are inconclusive. The study of working-age participants in Finland [55] showed that self-reported stress was associated with objective physiological stress, although the Finish study participants were affected by different factors. Therefore, a combination of both self-report and objective measures would be optimal in order to assess stress most accurately.

Also, it is difficult to derive causal relationships among perceived stress, occupational groups and sedentary behaviour from a cross-sectional analysis. Other researchers in similar studies have used 3 different occupational categories—white-collar, blue-collar and, in addition, professionals category [27]. Future studies involving more than two occupational categories could be beneficial.

## 5. Conclusions

This study provides current evidence of the role of sedentary behaviour on perceived stress among white-collar and blue-collar workers. Since these are the two largest occupational groups in our society, comparing workers of these occupations we were able to assess the most significant differences in the level of perceived stress. Our findings revealed that we, as a working society, have to manage two main aspects: white-collar workers should try to improve their physical activity, especially by changing sedentary behaviour to something more active in order to control and reduce high stress and its consequences. Blue-collar workers should try to avoid unhealthy lifestyle habits and to manage not only psychological, but also physical work stressors. 

Sedentary behaviour does not impact blue-collar workers as much as white-collar workers in terms of high perceived stress, although overall blue-collar workers are more prone to higher levels of perceived stress. Since their occupation requires a more active outcome, sedentary behaviour does not have as significant an impact on blue-collar workers as it does on white-collar workers. It is considered that blue-collars are more socially vulnerable due to their occupational position and lifestyle challenges, while white-collar workers, especially males, suffer more from work stress due to their positional requirements and the importance of their decisions.

The strongest associations in this study was between high perceived stress and HP, chronic diseases, low levels of PA and overweight/obesity. Controlling these risk factors could lead to better stress management. Further studies could be accomplished by including and comparing more occupational groups, as listed in ISCO. Providing objective measures to evaluate physical activity (pedometers, activity/blood pressure monitors, accelerometers, etc.,) could help derive more accurate and reliable results.

## Figures and Tables

**Table 1:** The characteristics of study participants by occupational groups.

Variable	Occupational Group		*p*-value	Correlation
	White-Collar *n* (%)	Blue-Collar *n* (%)		
**Age groups**				
≤30	89 (20.8)	20 (14.0)	0.002	0.163 ^a^
31–45	189 (44.2)	47 (32.9)		
46–60	132 (30.8)	65 (45.5)		
≥61	18 (4.2)	11 (7.7)		
**Gender**			0.000	0.462 ^b^
Women	279 (65.2)	17 (11.9)		
Men	149 (34.8)	126 (88.1)		
**Educational level**			0.000	0.477 ^a^
Low	70 (16.4)	92 (64.3)		
Medium	126 (29.4)	32 (22.4)		
High	232 (54.2)	19 (13.33)		
**Marital status**			0.942	0.026 ^a^
Married	292 (68.2)	101 (70.6)		
Divorced	54 (12.6)	16 (11.2)		
Single	74 (17.3)	23 (16.1)		
Widowed	8 (1.9)	3 (2.1)		
**Work PA**			0.000	0.334 ^a^
Low	141 (32.9)	22 (15.4)		
Moderate	182 (42.5)	34 (23.8)		
High	105 (24.5)	87 (60.6)		
**Leisure PA**			0.001	0.162 ^a^
Low	100 (23.4)	51 (35.7)		
Moderate	287 (67.1)	70 (49.0)		
High	41 (9.6)	22 (15.4)		
**PA (min/week)**				0.068 ^a^
≤150	405 (94.6)	140 (97.6)	0.104	
>150	23 (5.4)	3 (2.1)		
**Sedentary behaviour**			0.205	0.053 ^a^
<median	335 (78.3)	119 (83.2)		
≥median	93 (21.7)	24 (16.8)		
**Income (Eur)**			0.006	0.129 ^a^
≤1000	148 (43.8)	36 (29.5)		
>1000	190 (56.2)	86 (70.5)		
**Smoking**			0.000	0.385 ^b^
No	320 (74.8)	46 (32.2)		
Yes	108 (25.2)	97 (67.8)		
**BMI**			0.000	0.188 ^a^
Normal	176 (49.0)	28 (26.7)		
Overweight/obesity	183 (51.0)	77 (73.3)		
**Chronic disease**			0.165	0.056 ^b^
No	351 (82.0)	110 (76.9)		
Yes	77 (18.0)	33 (23.1)		
**HP**			0.165	0.058 ^b^
No	386 (90.2)	123 (86.0)		
Yes	42 (9.8)	20 (14.0)		
**Children (<18 years)**			0.824	0.009 ^b^
No	241 (56.3)	79 (55.2)		
Yes	187 (43.7)	64 (44.8)		
**Walkers**			0.868	−0.007 ^b^
No	418 (97.7)	140 (97.9)		
Yes	10 (2.3)	3 (2.1)		
**Public transport users**			0.000	−0.161 ^b^
No	291 (68.0)	121 (84.6)		
Yes	137 (32.0)	22 (15.4)		
**Bicycle users**			0.200	−0.054 ^b^
No	413 (96.5)	141 (98.6)		
Yes	15 (3.5)	2 (1.4)		
**Car users**			0.006	0.114 ^b^
No	128 (29.9)	26 (18.2)		
Yes	300 (70.1)	117 (81.8)		
**Stress**				
Low perceived stress	201 (47.0)	52 (36.4)	0.027	0.092 ^b^
High perceived stress	227 (53.0)	91 (63.6)		

*p* values for the Chi-square test. ^a^ Cramer’s V correlation coefficient; ^b^ the phi correlation coefficient.

**Table 2:** The relationship between the potential risk factors and perceived stress level.

Variable	Stress		OR (95% CI)
	Low Perceived Stress *n* (%)	High Perceived Stress *n* (%)	
**Age groups**			
≤30	68 (26.9)	41 (12.9)	1
31–45	99 (39.1)	137 (43.1)	2.30 *** (1.44–3.66)
46–60	76 (30.0)	121 (38.1)	2.64 *** (1.63–4.28)
≥61	10 (4.0)	19 (6.0)	3.15 ** (1.34–7.43)
**Gender**			
Women	127 (50.2)	169 (53.1)	1.13 (0.81–1.57)
Men	126 (49.8)	149 (46.9)	1
**Educational level**			
Low	65 (25.7)	97 (30.5)	1.46 (0.98–2.17)
Medium	64 (25.3)	94 (29.6)	1.43 (0.96–2.15)
High	124 (49.0)	127 (39.9)	1
**Marital status**			
Married	176 (69.6)	217 (68.2)	1
Divorced	26 (10.3)	44 (13.8)	1.37 (0.81–2.32)
Single	49 (19.4)	48 (15.1)	0.79 (0.51–1.24)
Widowed	2 (0.8)	9 (2.8)	3.65 (0.78–17.11)
**Work PA**			
Low	61 (24.1)	102 (32.1)	1.00 (0.65–1.54)
Moderate	120 (47.4)	96 (30.2)	0.48 *** (0.32–0.71)
High	72 (28.5)	120 (37.7)	1
**Leisure PA**			
Low	72 (28.5)	79 (24.8)	1.13 (0.63–2.04)
Moderate	149 (58.9)	208 (65.4)	1.44 (0.84–2.47)
High	32 (12.6)	31 (9.7)	1
**PA (min/week)**			
≤150	235 (92.9)	310 (97.5)	2.97 * (1.27–6.94)
>150	18 (7.1)	8 (2.5)	1
**Sedentary behaviour**			
<median	207 (81.8)	247 (77.7)	1
≥median	46 (18.2)	71 (22.3)	1.29 (0.85–1.96)
**Income (Eur)**			
≤1000	78 (40.2)	106 (39.8)	1
>1000	116 (59.8)	160 (60.2)	1.02 (0.70–1.48)
**Smoking**			
No	173 (68.4)	193 (60.7)	1
Yes	80 (31.6)	125 (39.3)	1.40 (0.99–1.98)
**BMI**			
Normal	106 (58.2)	98 (34.8)	1
Overweight/obesity	76 (41.8)	184 (65.2)	2.62 *** (1.79–3.84)
**Chronic disease**			
No	229 (90.5)	232 (73.0)	1
Yes	24 (9.5)	86 (27.0)	3.53 *** (2.17–5.76)
**HP**			
No	246 (97.2)	263 (82.7)	1
Yes	7 (2.8)	55 (17.3)	7.35 *** (3.28–16.45)
**Children (<18 y)**			
No	148 (58.5)	172 (54.1)	1
Yes	105 (41.5)	146 (45.9)	1.20 (0.86–1.67)
**Walkers**			
No	245 (96.8)	313 (98.4)	1
Yes	8 (3.2)	5 (1.6)	0.49 (0.16–1.51)
**Public transport users**			
No	180 (71.1)	232 (73.0)	1
Yes	73 (28.9)	86 (27.0)	0.91 (0.63–1.32)
**Bicycle users**			
No	241 (95.3)	313 (98.4)	1
Yes	12 (4.7)	5 (1.6)	0.32 * (0.11–0.92)
**Car users**			
No	66 (26.1)	88 (27.7)	1
Yes	187 (73.9)	230 (72.3)	0.92 (0.64–1.34)
**Occupational group**			
White-collar	201 (79.4)	227 (71.4)	1
Blue-collar	52 (20.6)	91 (28.6)	1.55 * (1.05–2.29)

* *p*-value < 0.05, ** *p*-value < 0.01, *** *p*-value < 0.001.

**Table 3:** Odds ratio for high perceived stress by hours of sedentary behaviour according to occupational groups and gender.

Variables	Crude OR (95% CI)	aOR (95% CI)
**White-collar**		
Sedentary behaviour 3 h and more per day	1.63 * (1.02–2.61)	2.93 ** (1.48–5.81) ^a^
Women		
Sedentary behaviour 3 h and more per day	1.93 (0.99–3.75)	3.26 ** (1.23–8.65) ^b^
Men		
Sedentary behaviour 3 h and more per day	1.58 (0.78–3.19)	4.34 ** (1.46–12.95) ^b^
**Blue-collar**		
Sedentary behaviour 3 h and more per day	0.62 (0.26–1.51)	1.25 (0.23–6.81) ^c^
Women		
Sedentary behaviour 3 h and more per day	0.18 (0.02–2.15)	0.12 (0.01–2.74) ^d^
Men		
Sedentary behaviour 3 h and more per day	0.71 (0.27–1.87)	0.69 (0.24–1.98) ^e^

* *p*-value < 0.05, ** *p*-value < 0.01; ^a^ adjusted for: gender, income, children (<18 y), BMI and work PA; ^b^ adjusted for: income, children (<18 years), BMI and work PA; ^c^ adjusted for: gender, children (<18 y), BMI and work PA; ^d^ adjusted for: marital status and smoking; ^e^ adjusted for: marital status, smoking and chronic diseases.
